# TEAD1 and c-Cbl are novel prostate basal cell markers that correlate with poor clinical outcome in prostate cancer

**DOI:** 10.1038/sj.bjc.6604774

**Published:** 2008-11-11

**Authors:** J F Knight, C J Shepherd, S Rizzo, D Brewer, S Jhavar, A R Dodson, C S Cooper, R Eeles, A Falconer, G Kovacs, M D Garrett, A R Norman, J Shipley, D L Hudson

**Affiliations:** 1 Department of Molecular Carcinogenesis, The Bob Champion Prostate Stem Cell Team, The Institute of Cancer Research, Sutton, Surrey SM2 5NG, UK; 2 Department of Molecular Carcinogenesis, Cell Transformation Team, The Institute of Cancer Research, Sutton, Surrey SM2 5NG, UK; 3 Translational Cancer Genetics, The Institute of Cancer Research, Sutton, Surrey SM2 5NG, UK; 4 Department of Pathology and Molecular Genetics, University of Liverpool, Duncan Building, Liverpool, UK; 5 Department of Oncology, Charing Cross Hospital, London W6 8RF, UK; 6 Ruprecht-Karls-Universitat, Heidelberg Klinikum, Molekular Onkologie, Im Neuenheimer Feld 365, Heidelberg 69120, Germany; 7 Cancer Research UK Centre for Cancer Therapeutics, The Institute of Cancer Research, Sutton, Surrey SM2 5NG, UK; 8 Department of Medical Statistics, Royal Marsden NHS Trust, Sutton, Surrey SM2 5NG, UK; 9 Department of Molecular Carcinogenesis, The Institute of Cancer Research, Sutton, Surrey SM2 5NG, UK

**Keywords:** prostate cancer, c-Cbl, TEAD1, differentiation, laser-capture microdissection

## Abstract

Prostate cancer is the most frequently diagnosed male cancer, and its clinical outcome is difficult to predict. The disease may involve the inappropriate expression of genes that normally control the proliferation of epithelial cells in the basal layer and their differentiation into luminal cells. Our aim was to identify novel basal cell markers and assess their prognostic and functional significance in prostate cancer. RNA from basal and luminal cells isolated from benign tissue by immunoguided laser-capture microdissection was subjected to expression profiling. We identified 112 and 267 genes defining basal and luminal populations, respectively. The transcription factor TEAD1 and the ubiquitin ligase c-Cbl were identified as novel basal cell markers. Knockdown of either marker using siRNA in prostate cell lines led to decreased cell growth in PC3 and disrupted acinar formation in a 3D culture system of RWPE1. Analyses of prostate cancer tissue microarray staining established that increased protein levels of either marker were associated with decreased patient survival independent of other clinicopathological metrics. These data are consistent with basal features impacting on the development and clinical course of prostate cancers.

Prostate cancer is one of the most common malignancies in the western world, but markers that accurately predict the highly variable course of the disease are lacking.

Normal prostate tissue consists of glands containing two well-defined epithelial layers surrounding a central lumen. The outer basal layer is the site of proliferation (Hudson *et al*, 2001) and the inner layer is made up of secretory luminal cells arising from the basal layer through differentiation (Signoretti *et al*, 2005). Changes in the balance between epithelial cell division and differentiation may play a key role in the development of both prostate cancer and benign prostatic hyperplasia (BPH) (Bonkhoff and Remberger, 1996). Prostate cancer cells resemble those of the luminal layer (Browne *et al*, 2004), particularly in their expression of prostate-specific antigen (PSA) and the androgen receptor (AR). Unlike luminal cells, however, and in common with basal cells, cancer cells maintain the ability to proliferate. Although a defining feature of prostate cancer is the complete loss of the basal cell layer, there is evidence for a maintained expression of genes normally restricted to basal cells, including *MET* (Pisters *et al*, 1995), *BCL-2* (McDonnell *et al*, 1992), *SOX9* (Wang *et al*, 2007), *KLK4* (Klokk *et al*, 2007) and *S100A9* (Hermani *et al*, 2005). Furthermore, the cancer-initiating cell is widely believed to be of basal origin (Bui and Reiter, 1998), expressing known basal markers including keratin (K)14, *α*2*β*1 integrin and CD44 (Collins *et al*, 2005; Patrawala *et al*, 2007). We therefore hypothesised that basal cell features are of key importance in the development of prostate tumours.

To address this, we used immunoguided laser-capture microdissection to isolate prostate basal and luminal epithelial cells under conditions that preserve RNA of sufficient quality for expression profiling. This identified gene expression patterns relating to proliferation and differentiation within benign prostate epithelium. *TEAD1* (a transcription factor) and *c-Cbl* (an E3 ubiquitin ligase) were confirmed as novel basal cell markers. Analysis of their expression across a range of prostate cancer samples on tissue microarrays (TMAs) demonstrated each to have a prognostic value. Finally, the functional significance of TEAD1 and c-Cbl in controlling growth and differentiation of prostate cell lines was examined using RNA interference.

## Materials and methods

### Clinical material

Frozen tissue was obtained from five consented patients undergoing transurethral resection of the prostate for BPH.

The TMA consisted of 774 formalin-fixed paraffin-embedded cores representing benign epithelium (43 patients), hyperplastic epithelium (124 patients) and prostate cancer (147 patients) (Foster *et al*, 2004). The prostate cancer samples were from consecutive patients attending the Royal Marsden NHS Trust from 1992 with follow-up times of up to 16.6 years (median 6.9 years). Owing to the long follow-up period for this patient set, PSA values were unavailable for many patients.

### Immunohistochemistry and laser microdissection pressure catapulting

Basal cells were distinguished by immunostaining for K14 (Abd Serotec, Kidlington, UK). Primary and secondary antibodies were used at 1 : 25 and 1 : 100 dilutions, respectively, allowing 5-min incubations. Antibodies were visualised using the Vectastain Elite ABC kit and chromogen 3,3′-diaminobenzidine (DAB; Vector Laboratories Ltd, Peterborough, UK) with incubations of 5 and 2 min, respectively, and a 7 s haematoxylin counterstain. Sections were rapidly dehydrated through graded alcohols and then air-dried for 5 min. RNase inhibitor (Roche Diagnostics Ltd, Burgess Hill, UK) was used at a concentration of 0.5 U *μ*l^−1^ in all solutions.

Microdissection was performed using the PALM Microbeam (PALM Microlaser Technologies GmbH, Bernried, Germany) using the ‘close-cut autoLPC’ function. Capture buffer consisted of 0.5% Igepal (Sigma-Aldrich, Poole, UK) in HPLC water with RNase inhibitor. A total of 100–500 cells were captured from each section and cells from two adjacent sections were pooled.

### RNA extraction and amplification

The Picopure RNA extraction kit (Molecular Devices Ltd, Wokingham, UK) was used according to the manufacturer's protocol. RNA was amplified through two rounds using the ExpressArt Trinucleotide Amplification Nano Kit for degraded RNA (Amp Tec GmbH, Hamburg, Germany). Amplified RNA quality was assessed using the Agilent 2100 Bioanalyser (Agilent Technologies UK Ltd, South Queensferry, UK).

### Gene expression profiling and analysis

The Cancer Research UK Human Whole Genome-wide cDNA Array v1.0.0 (32K) (http://www.crukdmf.icr.ac.uk) was used as described earlier (Shepherd *et al*, 2008) and further information is given in Supplementary Information. Data were normalised and filtered to generate lists of genes with minimum 1.4-fold change in expression in at least three of five samples. The functional significance of differentially expressed genes was analysed using the ‘Database for Annotation Visualization and Integrated Discovery’ (DAVID) (http://david.abcc.ncifcrf.gov).

### Semiquantitative PCR

Amplified RNA was reverse transcribed using Superscript II (Invitrogen Ltd, Paisley, UK). Primer sequences and cycling conditions are provided in Supplementary Information. Following two rounds of PCR, products were resolved on a 3% agarose gel and detected under a UV transilluminator (BioDoc-It system; UVP, Cambridge, UK).

### Immunofluorescence and immunohistochemistry

Paraffin-embedded sections were microwaved in antigen-unmasking solution (Vector Laboratories) for 30 min. Antibodies used are listed in Supplementary Table S1. Immunofluorescent labelling was carried out as described earlier (Alam *et al*, 2004). Immunohistochemical staining of TMA cores was detected using the Advance HRP kit and Liquid DAB+ substrate chromogen system (Dako, Ely, UK). Stained cores were reviewed by a pathologist. c-Cbl staining was scored on a scale of increasing intensity (1–3) and TEAD1 by increasing intensity (0–3) and as diffuse (in all tumour cells) or focal (localised to small groups of tumour cells).

### Statistical analyses for survival

Survival was measured using the dates of death from any cause and death from prostate cancer. Patients were censored on their date of last follow-up if no death had been recorded. Lifetables were generated using the models of Kaplan and Meier. Differences between groups were examined using the log-rank test. Univariate and multivariate Cox regression analysis was performed using stepwise methods, a *P*-value of 0.05 was used as a threshold for inclusion in the model.

### Cell culture

The RWPE1 cell line was derived from benign prostate and PC3 from a prostate cancer bone metastasis (both ATCC, LGC Standards, Teddington, UK). PC3 cells were cultured in F-12 Ham medium (Sigma-Aldrich) with 10% FCS (PAA Laboratories, Yeovil, UK) and L-glutamine (Invitrogen). RWPE1 cells were cultured in keratinocyte serum-free medium (KSFM) supplemented with bovine pituitary extract (25 mg), human recombinant epidermal growth factor (EGF) (2.5 *μ*g) and L-glutamine (Invitrogen). Supplemented KSFM is referred to as bKSFM.

### siRNA transfections

Pre-designed siRNA oligonucleotide duplexes for TEAD1 and c-Cbl were purchased from Qiagen (Crawley, UK) and ON-TARGET*plus* non-targeting siRNA no. 2 from Dharmacon (Thermo Scientific, Cramlington, UK). Mock controls were included. Reverse transfections were performed by mixing suspended cells with the siRNA oligonucleotides, Optimem I (Invitrogen) and Hiperfect (Qiagen), before plating. Final siRNA oligonucleotide concentrations were 5 nM (RWPE1) and 10 nM (PC3).

### MTS proliferation assays

Reverse-transfected cells were plated into 96-well plates or 6 cm dishes. At each time point, proliferation was assessed using the Cell Titre 96 Aqueous One MTS assay (Promega, Southampton, UK). Plates were read using a BioTek ELx800 plate reader at 490 nm. Dishes were incubated for 72 h and protein extracted for knockdown verification. A one-way ANOVA with a Dunnett's multiple comparison test was performed in GraphPad Prism Version 5.01 (GraphPad Software Inc.).

### 3D culture in matrigel

Twenty-four hours after reverse transfection, RWPE1 cells were resuspended in bKSFM containing 2% Matrigel (BD Biosciences, Oxford, UK) and seeded into Matrigel-coated eight-well chamber slides (Nunc, VWR, Lutterworth, UK) (Debnath *et al*, 2003). Duplicate dishes were incubated for 120 h (corresponding to day 4 of the matrigel assay) before protein extraction. Acini formed by day 4 were scored and photographed under phase-contrast microscopy. The numbers of organised acini and disorganised aggregates were recorded for five fields of view. The number of disorganised aggregates was expressed as a percentage of the total number of structures and referred to as the percentage failure of acini formation.

### Western blotting

Protein was extracted using mammalian protein extraction reagent (Pierce, Thermo Scientific, Cramlington, UK) supplemented with EDTA and protease inhibitor cocktail (Pierce). Lysates were resolved on a 4–12% Bis–Tris Nupage gel (Invitrogen). Antibodies were used as follows: TEAD1 (1 : 850), c-Cbl (1 : 1000), GAPDH (Chemicon, Chandlers Ford, UK) 1 : 10 000 and ECL peroxidase secondary antibody (GE Healthcare, Amersham, UK) (1 : 5000).

## Results

### Expression profiling of microdissected basal and luminal cells identifies new markers for both populations

**Figure 1:**
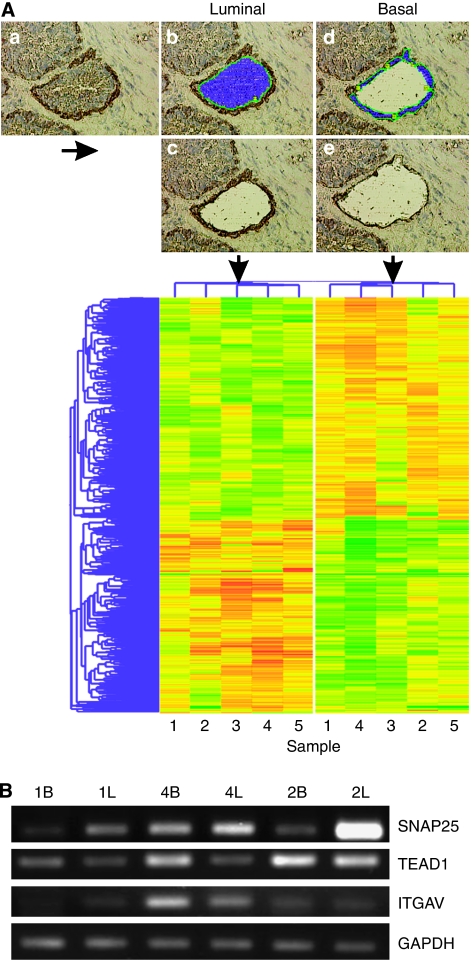
Laser-capture microdissection pressure catapulting (LMPC) and expression profiling of basal and luminal prostate epithelial cells. (**A**) Snap-frozen BPH tissue was rapid immunostained for basal cell marker K14, with nuclei counter stained with haematoxylin (**a**). Basal and luminal epithelial cells were laser captured sequentially; luminal cells were selected (**b**), then captured (**c**), followed by selection (**d**) and capture (**e**) of K14-positive basal cells. Extracted RNA from five patients was used for expression profiling by cDNA microarray. Differentially expressed genes were identified through ANOVA (*P*=0.05). Genes were entered into hierarchical cluster analysis represented here by a dendrogram. (**B**) Semiquantitative RT–PCR of amplified RNA from three patient samples (1, 2 and 4) confirming differential basal (B) and luminal (L) expression of SNAP25, TEAD1 and integrin *α*V. Luminal expression of SNAP25 was confirmed in three out of three patients; basal expression of TEAD1 was confirmed in two out of three patients and basal expression of integrin *α*V was confirmed in one out of three patients.

Basal and luminal cells were laser captured as illustrated in Figure 1A. Two rounds of RNA amplification yielded 24–38 *μ*g of RNA with fragment sizes ⩽1500 bp. Microarray hybridisations were performed for each of the five basal and luminal samples, hybridising each against universal reference cDNA. Hierarchical clustering of the gene set (Figure 1A) showed a clear segregation of basal and luminal samples. A 1.4-fold cutoff relative to reference cDNA was applied for all differentially expressed genes, yielding basal and luminal lists of 112 and 267 genes, respectively. Basal/luminal or luminal/basal fold-change ratios were calculated to directly compare the two populations (Supplementary Tables S2a and S2b). K14 was overexpressed in the basal population, confirming positive cell separation. A number of differentially expressed genes were selected for verification of the microarray data by semiquantitative PCR (Figure 1B). Luminal expression of SNAP25 was confirmed in three out of three patients and basal expression of TEAD1 was confirmed in two out of three patients. Basal expression of integrin *α*V was confirmed in just one out of three patients and c-Cbl failed to generate any product. Owing to the varying success of the PCR, due, most likely, to the poor quality of the starting RNA, all further localisation confirmation was carried out using antibody staining.

To identify differences in key biological processes between basal and luminal cells, the web-based ontology program DAVID was used. This program categorises genes based on their biological or molecular function and assigns a *P*-value to each category. Filtering on a *P*-value less than or equal to 0.05 returned 14 basal cell categories and 23 luminal categories (Supplementary Table S3).

### Immunofluorescence confirms the localisation of selected markers

**Figure 2:**
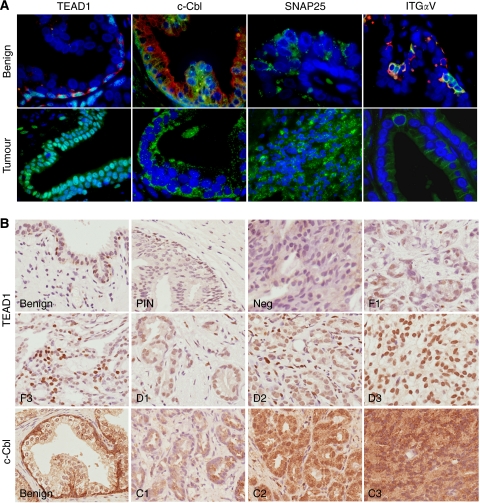
Immunofluorescent and immunohistochemical labelling of benign and cancerous prostate tissue for basal and luminal cell markers. (**A**) Benign tissue: nuclear TEAD1 labelling (green) was found in K14-positive basal cells (red). Note turquoise double-stained TEAD1-positive nuclei. Cytoplasmic staining for c-Cbl (green) was strong in basal cells and weak in luminal cells as shown by co-labelling with luminal marker K8 (red). SNAP25 (green) staining was luminal in a speckled vesicle-like pattern. Integrin *α*V (green) was restricted to basal cells, either co-localised to K14 (red) or alone. In prostate tumours, the expression of TEAD1 and c-Cbl was strong despite the absence of a basal layer. Occasional areas of tumour tissue labelled intensely for SNAP25. Integrin *α*V expression in tumour tissue was extremely weak. Original magnification × 63 to × 100. (**B**) Scoring systems were derived for both TEAD1 and c-Cbl. TEAD1 scored first as focal (F) or diffuse (D) followed by a score for intensity (1=low to 3=high). Tumour cores lacking TEAD1 expression were scored as negative (neg). Scoring for c-Cbl was based on intensity alone (C1=low to C 3=high). Original magnification × 40.

Commercially available antibodies were used to confirm the protein localisation of a selection of novel markers in benign and malignant prostate tissue by immunofluorescence. The selection included genes identified as basal (*ITGAV*, *TEAD1*, *c-Cbl* and *IL6*) and luminal (*SPRY1* and *SNAP25*). Double labelling with either K14 or K8 was used to confirm basal or luminal localisation of staining within benign prostate tissue. In agreement with the expression profiling data, TEAD1, c-Cbl and integrin *α*V were expressed in the basal layer and SNAP25 in the luminal layer (Figure 2A). We were unable to detect staining for IL6 or SPRY1. TEAD1 staining was nuclear in all cells within the basal layer, whereas c-Cbl was strongly cytoplasmic in the basal layer, with weak luminal staining. Integrin *α*V was found in subsets of cells within the basal layer, and clusters of integrin *α*V-positive cells were either K14 positive or negative. The SNAP25 antibody detected luminal cells, with a vesicular staining pattern. The same antibodies were also used on a small selection of prostate tumour samples. Although expression of SNAP25 and integrin *α*V could be detected in the tumours, integrin *α*V labelling was often weak, and results for SNAP25 were inconsistent between samples. As c-Cbl and TEAD1 staining was consistently high, these proteins were chosen for further study. c-Cbl was expressed in the cytoplasm of epithelial tumour cells, whereas TEAD1 was confined to the nuclei (Figure 2A).

### TEAD1 and c-Cbl are independent prognostic factors of prostate cancer

**Figure 3:**
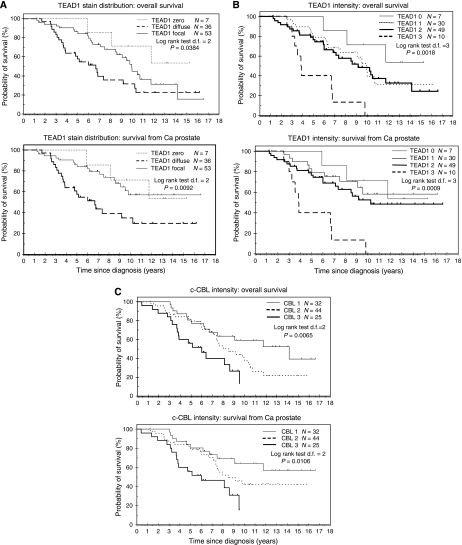
Kaplan–Meier curves. Kaplan–Meier analysis of overall and disease-specific survival for prostate cancers stained for TEAD1 (**A** and **B**) and c-Cbl (**C**). TEAD1 staining scored for either distribution (**A**) or intensity (**B**).

To determine the clinical significance of TEAD1 and c-Cbl expression in prostate tumours, TMAs were stained for the two markers. Staining and patient data for positive cores are given in Table 1, with examples of typical staining patterns in Figure 2B. As shown by immunofluorescent staining, TEAD1 was localised to basal cell nuclei, whereas that for c-Cbl was cytoplasmic. In some strongly stained regions of the TMA, c-Cbl also appeared to be perinuclear (Figure 2B C2). The scoring systems are described in the Materials and Methods. Kaplan–Meier survival curves for TEAD1 and c-Cbl, representing deaths from all causes and prostate cancer-specific deaths, are shown in Figure 3. Staining distribution (focal or diffuse) and staining intensity for TEAD1 were considered as separate parameters. Diffuse staining was found to correlate with a poorer patient prognosis than focal (*P*=0.0092) (Figure 3A). However, this was not a significant factor in multivariate analysis because of a strong correlation with Gleason score, with diffuse TEAD1 staining found in predominately high Gleason cancers (Table 1). Staining intensity for TEAD1 showed a clear overlap in patient survival for groups 1 and 2, and patients with the highest intensity staining (group 3) had a highly significant reduced survival rate (*P*=0.0009) (Figure 3B). For both parameters, these differences are most striking in the prostate cancer-specific survival data.

c-Cbl staining was scored solely by intensity (scores 1–3). Survival data showed a clear divergence of intensity groups 1 and 2 at 7.5 years post-diagnosis, with an increased mortality rate for group 2 (Figure 3C). Group 3 patients had a particularly poor prognosis with a 50% survival rate of only 6 years compared with 14 years for group 1 (*P*=0.0106).

In Cox regression multivariate analysis, TEAD1 staining intensity scores were significantly associated with increased mortality, with a hazard ratio of 1.56 (95% CI 1.02–2.37; *P*=0.037) (Table 2).

Similarly, c-Cbl was found to be a highly significant independent predictor of survival (*P*=0.0005) with a hazard ratio of 1.99 (95% CI 1.35–2.92). One factor significant in the univariate analysis, although not in the multivariate analysis, was age.

Interestingly, of the 10 patients presenting with TEAD1 level 3, only four had level-3 c-Cbl, and there does not therefore appear to be a direct correlation between the two markers. This suggests that while high levels of either TEAD1 or c-Cbl are indicative of equally poor prognosis, they may identify somewhat different tumour types, supporting the observation that prostate cancer is a truly heterogeneous disease.

### Knockdown of TEAD1 or c-Cbl reduces proliferation in PC3 cells

**Figure 4:**
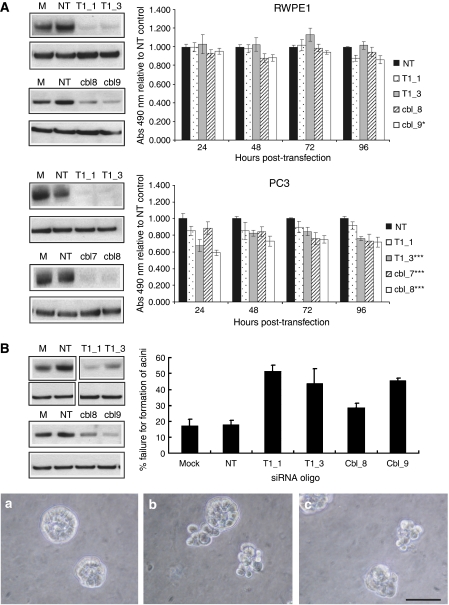
The effects of siRNA-mediated knockdown of TEAD1 or c-Cbl in RWPE1 and PC3 cell lines. RWPE1 and PC3 cells were reverse transfected using two siRNA oligos per target gene. Mock transfection (M) and non-targeting siRNA (NT) were controls. (**A**) MTS proliferation assays were performed over 96 h post-transfection. Proliferation was normalised to that of the non-targeting control. Mean of three experiments, bars=s.e.m.; ^*^P<0.05; ^***^P<0.01. Representative western blots at 72 h post-transfection are shown. (**B**) Transfected RWPE1 cells were grown in Matrigel for 4 days and the % failure for the formation of spherically polarised acini was calculated. Mean of three experiments, bars=s.e.m. Knockdown of TEAD1 and c-Cbl significantly increased the failure rate (*P*-values; T1_1=0.006, T1_3=0.009; cbl_8=0.08, cbl_9=0.003). Phase-contrast microscopy images of spheres: A=non-targeting control, B=T1_1 and C=cbl_9. Original magnification × 40, bar=50 *μ*m. The level of knockdown remaining after 4 days was confirmed by western blotting. Representative blots for the three experiments are shown.

To investigate whether TEAD1 or c-Cbl has a role in proliferation, two well-established prostate cell lines were transfected with siRNA oligonucleotides targeting each gene. Knockdown was confirmed by western blotting and the effect on proliferation quantitated by MTS assays carried out over a 96-h time course (Figure 4A). Although the proliferation of RWPE1 cells was not affected by knockdown of either TEAD1 or c-Cbl, the proliferation of PC3 cells was significantly reduced. Data were normalised to proliferation of cells transfected with non-targeting siRNA. Normalisation to proliferation of mock-transfected cells yielded similar results.

### Knockdown of TEAD1 or c-Cbl affects acinar formation in 3D culture

RWPE1 cells form spherically polarised acini, representative of the glandular structure of the prostate when cultured on matrigel (Webber *et al*, 1997). Cells were seeded in matrigel 24 h after transfection with TEAD1 or c-Cbl siRNA. After 4 days, acinar-like spherical structures with a well-defined border, often with less-dense cells in the centre, were formed (Figure 4Ba). Disorganised clusters were defined as aggregates of four or more cells that failed to form spherically polarised acini (Figure 4Bb and c). At day 4, the percentage failure of acini formation was calculated. The mean results of three independent experiments are shown in Figure 4B. TEAD1 knockdown significantly disrupted the number of spherically organised structures in comparison to the non-targeting control transfections, with an increased occurrence of disorganised clusters (T1_1=*P*0.006, T1_3=*P*0.009). Similarly, knockdown of c-Cbl also significantly reduced acini formation (cbl_8=*P*0.08, cbl_9=*P*0.003). These results are comparable with those of Kawano *et al* (2006), who found a 30% failure of acinar formation in response to the knockdown of the Wnt signalling pathway component *Dickkopf-3 (DKK3)*.

## Discussion

Deregulation of proliferation and differentiation of prostate epithelial cells are predicted to underlie the development and progression of prostate cancers. As these processes are not well understood, it is not currently possible to predict patient outcome accurately. To identify key molecular factors involved in these processes, we have profiled gene expression associated with the basal and luminal epithelial cell layers. Genes expressed in basal cells are involved in maintaining a proliferative undifferentiated phenotype and promote cell survival. Therefore, we hypothesised that genes with their expression normally restricted to the basal layer play a key role in the progression of prostate malignancy. In this study, we determined the prognostic significance in prostate cancer of two novel markers of benign prostate basal cells.

Approaches to isolating different epithelial cell populations from the prostate have included lengthy processing and cell culture. This is expected to alter patterns of gene expression compared with cells *in vivo*. Therefore, we used laser-capture microdissection (Espina *et al*, 2007) combined with immunostaining for cellular recognition. As standard immunohistochemistry protocols lower RNA quality (Fend *et al*, 1999), we optimised a protocol to minimise the K14 staining procedure, enabling expression profiling of the microdissected cells. The 1.46-fold elevation of K14 expression levels in basal cells is modest considering that staining patterns show basal specificity. However, a lack of direct concordance between relative mRNA and protein levels in prostate tissue has been demonstrated earlier (Pascal *et al*, 2008).

Gene ontology analysis defined the functional categories upregulated in the two cellular populations. Luminal cells expressed negative regulators of the cell cycle, including the cyclin-dependent kinase inhibitors *p21*^*CIP1/WAF1*^ (*CDKN1A*) and p*27*^*KIP*^ (*CDKN1B*), the latter having earlier been localised to prostate luminal cells (Harper *et al*, 1993; Fernandez *et al*, 1999), and the tumour suppressor genes *NBL1* (Enomoto *et al*, 1994), *NDRG1* (Daly-Burns *et al*, 2007) and *PHB*, which are downregulated in prostate cancer cells (Gamble *et al*, 2007). In keeping with the known interaction between the AR and calmodulin (Cifuentes *et al*, 2004), a further luminal functional category included several proteins related to calmodulin binding. Present in several functional categories was the luminal gene *SNAP25*. Although *SNAP25* has not earlier been described in prostate, it has an established role in membrane trafficking and exocytosis for hormone secretion in other tissues, such as the pancreas (Sadoul *et al*, 1995).

The basal cell profile was consistent with a proliferative phenotype, with expression of *CXCL1*, *NBS1* and *RPA*, all of which encode proteins shown earlier to promote proliferation (Chiang *et al*, 2003; Li *et al*, 2004; Givalos *et al*, 2007). The expression of *α*V *integrin* has earlier been described in metastatic prostate cancer (McCabe *et al*, 2007) but not in benign prostate cells, where it may have a role in adhesion to the basement membrane.

The protein localisation of two highly ranking genes in the basal list, protooncogene *c-Cbl* and the transcription factor *TEAD1*, confirmed them as basal cell markers not described earlier in prostate. Furthermore, an increased staining intensity for both c-Cbl and TEAD1 was observed in prostate cancer samples on a TMA. Significantly, multivariate analysis confirmed both as prognostic markers. Further study is now needed to investigate their possible clinical applications in the future. There is an urgent need for new predictive markers that distinguish the most aggressive prostate cancers at an early stage. Currently under investigation for its potential as a prognostic marker is the non-coding RNA PCA3 (Hessels *et al*, 2003). Although PCA3 has been proposed as an alternative to PSA for prostate cancer detection, its actual prognostic value is uncertain (van Gils *et al*, 2008). Likewise, although *α*-methylacyl CoA racemase (AMACR) staining is used diagnostically, it is not used to predict patient outcome (Luo *et al*, 2002). One recently discovered DNA biomarker that may provide prognostic information is the fusion between the gene *TMPRSS2* and the *ETS* family genes such as *ERG* (Tomlins *et al*, 2005). In particular, duplication of the *TMPRSS2–ERG* fusion has been shown to identify aggressive tumours in patients whose prostate cancers had been assigned only intermediate Gleason scores (Attard *et al*, 2008). As staining intensities of both TEAD1 and c-Cbl also predict survival independently of Gleason score, they may prove also to be of significant prognostic value.

*c-Cbl* belongs to a family of E3 ubiquitin ligases (Swaminathan and Tsygankov, 2006). It binds to and ubiquitinates a range of receptor tyrosine kinases including EGFR (de Melker *et al*, 2001) and MET (hepatocyte growth factor receptor) (Peschard *et al*, 2001), both of which are localised to the basal layer and have reported roles in prostate proliferation and differentiation (Ibrahim *et al*, 1993; Pisters *et al*, 1995; Knudsen and Edlund, 2004; Schlomm *et al*, 2007). Receptor tyrosine kinases bound to c-Cbl are targeted to the endosomal compartment from where they are either trafficked to lysosomes for degradation or recycled back to the plasma membrane (Thien and Langdon, 2001). c-Cbl is, therefore, normally regarded as a negative regulator of proliferation. Interestingly, however, knockdown of c-Cbl in PC3 cells decreased proliferation. Importantly, c-Cbl also functions as an adaptor protein in signal transduction, for example downstream of integrins in which c-Cbl has been shown to interact with c-Src (Edick *et al*, 2007). Further investigation is needed to establish the role of c-Cbl in regulating prostate cancer cell proliferation.

The TEAD family of transcription factors, of which TEAD1 is the best characterised, regulate growth and differentiation in many tissues. This is exemplified by an important role for TEAD1 in embryonic development (Sawada *et al*, 2008; Zhang *et al*, 2008). TEAD1 binds several co-activators to stimulate the transcription of target genes (Gupta *et al*, 1997, 2001). Importantly, Yes-associated protein (YAP) is a TEAD1 co-activator localised to basal cell nuclei in normal prostate and overexpressed in prostate cancer (Zhao *et al*, 2007). TEAD1–YAP complexes promote the transcription of proliferative genes upon translocation of YAP to the nucleus, a process regulated by the Hippo pathway, which controls cell-contact inhibition of growth (Bandura and Edgar, 2008; Wu *et al*, 2008). As knockdown of TEAD1 led to a reduced proliferation in PC3 cells, it is possible that an increased expression of both TEAD1 and YAP could stimulate proliferation in prostate cancer.

The fact that knockdown of either TEAD1 or c-Cbl decreases the proliferation of PC3, but not RWPE1, is very interesting. This may reflect the different origins of the cell lines, RWPE1 having been immortalised from benign cells, whereas PC3 is of prostate cancer origin, representative of a highly aggressive, metastatic disease. As both TEAD1 and c-Cbl are highly expressed in aggressive prostate cancers, it could be possible that these aggressive cancers, and hence PC3, are dependent on these genes, directly or indirectly, for their proliferative capacity. Alternatively, RWPE1 proliferation may have been unaffected by TEAD1 or c-Cbl knockdown due to the immortalisation of this cell line with HPV18, which leads to the inactivation of p53 and Rb and, therefore, artificially high levels of proliferation. As a consequence of this, RWPE1 may have the ability to proliferate independently of TEAD1 or c-Cbl.

Although knockdown of TEAD1 or c-Cbl in RWPE1 did not affect proliferation in 2D culture, the ability of these cells to form acini in an established 3D assay (Bello-DeOcampo *et al*, 2001; Kawano *et al*, 2006) was compromised. These data suggest that TEAD1 and c-Cbl could have roles in regulating prostate epithelial cell differentiation and may have additional functions in epithelial morphogenesis, such as regulating adhesion to the basement membrane and associated signalling. Earlier studies have implicated a role for TEAD1 in the differentiation of keratinocytes (Takahashi *et al*, 1995) and skeletal muscle cells (Maeda *et al*, 2002), and a role for c-Cbl in maintaining an organised epithelium has been suggested earlier (Fournier *et al*, 2000). These preliminary functional studies clearly warrant further investigation into the roles of c-Cbl and TEAD1 in growth and differentiation in the prostate.

In summary, we have used a novel approach to identify two basal cell markers with high prognostic significance for prostate cancer. Interestingly, our preliminary functional study using well-established prostate cell lines has suggested a role for both genes in cellular proliferation and differentiation in the prostate, the two processes that are aberrantly regulated in cancer. Further study of TEAD1, c-Cbl and other basal markers may allow us to get an insight into the key molecular pathways that become disrupted in prostate malignancy.

## Figures and Tables

**Table 1:** Summary of patient details for c-Cbl and TEAD1 staining

	**Patient summary**			
	**c-Cbl**	**TEAD1**		
Total cancers	101	96		
Total benign	90	95		
Age range (years)	43–84	43–84		
Median age (years)	64	66		
Follow-up time (years)	0.4–16.6	1.25–16.6		
Median follow-up (years)	6.75	6.85		
				
*AJCC stage*				
I	2	2		
II	36	34		
III	24	24		
IV LA T4 or N1	16	16		
IV metastatic	23	20		
				
*Gleason grade*				
Well (2–4)	17	18		
Moderate (5–7)	54	50		
Poor (8–10)	29	26		
Missing	1	2		
				
**Staining summary**				
	**Total positive**	**Metastasis positive**	**Gleason<7**	**Gleason⩾7**
CBL 1	32	8 (25%)	22 (71%)	9 (29%)
CBL 2	44	9 (21%)	27 (61%)	17 (39%)
CBL 3	25	6 (24%)	11 (44%)	14 (56%)
TEAD1 0	7	1 (14%)	6 (86%)	1 (14%)
TEAD1 1	30	6 (24%)	22 (75%)	7 (25%)
TEAD1 2	49	10 (20%)	21 (44%)	27 (56%)
TEAD1 3	10	3 (30%)	2 (20%)	8 (80%)
TEAD1 focal	53	14 (26.5%)	40 (75.5%)	12 (22.6%)
TEAD1 diffuse	36	18 (50%)	11 (30.6%)	24 (66.7%)

**Table 2:** Analysis of overall survival

**Variable**	**Group**	***N***	**d.f.**	***P*-value**	**Hazard ratio**	**Lower 95% confidence interval**	**Upper 95% confidence interval**
*Univariate analysis*							
c-Cbl staining intensity	1	32	1		1		
	2	44	1	0.1049	1.7123	0.8939	3.2802
	3	25	1	0.0017	3.2627	1.5569	6.8375
c-Cbl staining intensity	Continuous	101	1	0.0019	1.8090	1.2448	2.6290
Age	Continuous	101	1	0.0002	1.0664	1.0304	1.1037
PSA	Continuous	41	1	0.0009	1.0044	1.0018	1.0070
Gleason score	Continuous	100	1	0.0000	1.3670	1.2048	1.5510
Gleason score	Gleason<7	60	1		1		
	Gleason⩾7	40	1	0.0000	4.1135	2.3917	7.0748
AJCC stage	1	2	1	0.2349	3.5565	0.4384	28.8493
	2	36	1		1		
	3	24	1	0.0067	3.2883	1.3912	7.7726
	4 M0	16	1	0.0005	4.7513	1.9644	11.4919
	4 M1	23	1	0.0000	9.0662	3.9812	20.6460
							
*Multivariate analysis*							
c-Cbl staining intensity	Continuous	100	1	0.0005	1.9895	1.3513	2.9291
Gleason score	Gleason<7	60			1		
	Gleason⩾7	40	1	0.0001	3.5851	1.9242	6.6795
AJCC stage	1	2	1	0.1715	4.3962	0.5265	36.7064
	2	36			1		
	3	23	1	0.4293	1.4602	0.5711	3.7333
	4 M0	16	1	0.0039	3.7042	1.5206	9.0236
	4 M1	23	1	0.0000	8.6743	3.6294	20.7316
Variables not in the model							
Age	Continuous	100	1	0.3219			
							
*Univariate analysis*							
TEAD1 staining intensity	0	7	1		1		
	1	30	1	0.2966	1.9435	0.5580	6.7693
	2	49	1	0.1981	2.1990	0.6624	7.3009
	3	10	1	0.0033	7.4536	1.9482	28.5157
TEAD1 staining intensity	Continuous	96	1	0.0065	1.7102	1.1621	2.5169
Age	Continuous	96	1	0.0004	1.0673	1.0293	1.1066
PSA	Continuous	38	1	0.0045	1.0048	1.0015	1.0081
Gleason score	Continuous	94	1	0.0000	1.3415	1.1784	1.5272
Gleason score	Gleason<7	57	1		1		
	Gleason⩾7	37	1	0.0000	4.2115	2.3747	7.4688
AJCC stage	1	2	1	0.1672	4.4911	0.5331	37.8367
	2	34	1		1		
	3	24	1	0.0022	4.4203	1.7105	11.4227
	4 M0	16	1	0.0005	5.7426	2.1493	15.3436
	4 M1	20	1	0.0000	12.0225	4.7692	30.3073
							
*Multivariate analysis*							
TEAD1 staining intensity	Continuous	94	1	0.0365	1.5615	1.0284	2.3708
Gleason score	Gleason<7	57	1		1		
	Gleason⩾7	37	1	0.0011	3.0741	1.5632	6.0455
AJCC stage	1	2	1	0.0536	8.5127	0.9677	74.8866
	2	34	1		1		
	3	23	1	0.0840	2.4744	0.8855	6.9144
	4 M0	16	1	0.0035	4.4048	1.6275	11.9214
	4 M1	19	1	0.0000	12.6098	4.7733	33.3118
Variables not in the model							
Age	Continuous	94	1	0.0646			

PSA=prostate-specific antigen.

Statistical tests were performed as described in Materials and Methods. For statistical analysis, the AJCC stage 4 tumours were divided into categories without (M0) or with (M1) distant metastases.
