# Supplementary material for: TEAD1 and c-Cbl are novel prostate basal cell markers that correlate with poor clinical outcome in prostate cancer
Source: Br J Cancer. 2008 Nov 11;99(11):1849–58. doi: 10.1038/sj.bjc.6604774 (PMC2600693; doi:10.1038/sj.bjc.6604774)
Supplement: Supplementary Table S1 [file 6604774x1.pdf]

Online only

Supplementary Table 1 – antibodies used in this study

Primary antibodies

Antigen/clone	Species/Isotype	Source	Dilution
CBL	Rabbit IgG	Abcam plc, Cambridge, UK	1:500
IL6 / BE8	Mouse IgG_1	Abcam	1:100
ITGAV / clone 21	Mouse IgG_1	BD Biosciences, Oxford, UK	1:500
Keratin 8 / M20	Mouse IgG_1	Abcam,	1:100
Keratin 14 / LL002	Mouse IgG_3	Serotec, Oxford, UK	1:100/1:50 (LCM)
SNAP25	Rabbit IgG	Lab Vision Ltd, Suffolk, UK	1:200
Sprouty 1 / H-120	Rabbit IgG	Santa Cruz, Insight Biotechnology, Middx, UK	1:50
TEAD1 / Clone 31	Mouse IgG_1	BD Biosciences	1:200

Secondary antibodies

Primary antibodies were detected with FITC- or TRITC- conjugated goat anti-mouse or rabbit secondary antibodies (Southern Biotechnology, Cambridge Biosciences, Cambridge, UK) at 1:100 dilution. For double staining, two primary antibodies were simultaneously applied and detected using different immunoglobulin isotype specific antibodies.
