# Supplementary material for: TEAD1 and c-Cbl are novel prostate basal cell markers that correlate with poor clinical outcome in prostate cancer
Source: Br J Cancer. 2008 Nov 11;99(11):1849–58. doi: 10.1038/sj.bjc.6604774 (PMC2600693; doi:10.1038/sj.bjc.6604774)
Supplement: Supplementary Table S2a [file 6604774x2.pdf]

Online Only
Supplementary Table S2a: All genes up-regulated in basal cells (ratio to luminal cell expression)

Rank	Symbol	Name	Ratio	p-value	Clone ID	LLID
1	PPM1B	Protein phosphatase 1B, magnesium-dependent, beta isoform	2.910	0.0498	262916	5495
2	ITGAV	Integrin, alpha V (vitronectin receptor, CD51)	2.509	0.0194	754736	3685
3	TEAD1	TEA domain family member 1 (SV40 transcriptional enhancer factor)	2.322	0.0012	1377100	7003
4	CXorf38	Chromosome X open reading frame 38	2.308	0.0435	2141223	159013
5	LY6H	Lymphocyte antigen 6 complex, locus H	2.264	0.0350	5199047	4062
6	DNM1L	Dynamin 1-like	2.172	0.0221	781051	10059
7	SNRPB2	Small nuclear ribonucleoprotein polypeptide B"	2.142	0.0107	149537	6629
8	RGS11	Regulator of G-protein signaling 11	2.134	0.0354	277917	8786
9	CAPN7	Calpain 7	2.056	0.0350	321523	23473
10	DNALI1	Dynein, axonemal, light intermediate chain 1	2.033	0.0446	782688	7802
11	SCN2B	Sodium channel, voltage-gated, type II, beta	2.025	0.0370	813628	6327
12	C1ORF19	Chromosome 1 open reading frame 19	1.994	0.0288	1588486	116461
13	CBL	Cas-Br-M (murine) ecotropic retroviral transforming sequence	1.986	0.0025	2310900	867
14	VEGFC	Vascular endothelial growth factor C	1.954	0.0181	44993	7424
15	C6ORF211	Chromosome 6 open reading frame 211	1.947	0.0277	785795	79624
16	PPP1R12C	Protein phosphatase 1, regulatory (inhibitor) subunit 12C	1.947	0.0456	193710	54776
17	CD58	CD58 molecule	1.930	0.0209	490368	965
18	TXNL4A	Thioredoxin-like 4A	1.923	0.0205	729914	10907
19	GDAP2	Ganglioside induced differentiation associated protein 2	1.917	0.0311	711847	54834
20	LIX1L	Lix1 homolog (mouse) like	1.910	0.0054	785256	128077
21	GLCCI1	Glucocorticoid induced transcript 1	1.890	0.0097	375839	113263
22	CPZ	Carboxypeptidase Z	1.876	0.0077	770462	8532
23	SKD3	ClpB caseinolytic peptidase B homolog (E.coli)	1.869	0.0459	274679	81570
24	A2M	Alpha-2-macroglobulin precursor	1.859	0.0242	44180	144571
25	SFRS1	Splicing factor, arginine/serine-rich 1 (splicing factor 2)	1.855	0.0383	80399	6426
26	TAF7	TATA box binding protein (TBP)-associated factor, 55kDa	1.850	0.0350	365930	6879
27	LOC220416	Similar to Leucine-rich repeat protein SHOC-2	1.839	0.0044	1618787	220416
28	KIFAP3	Kinesin-associated protein 3	1.821	0.0340	28810	22920
29	BOMB	WW and C2 domain containing 2	1.818	0.0136	22216	80014
30	ARL1	ADP-ribosylation factor-like 1	1.814	0.0154	283034	400
31	RPA1	Replication protein A1, 70kDa	1.808	0.0228	796096	6117
32	C9ORF102	Chromosome 9 open reading frame 102	1.806	0.0083	259134	56959
33	DCTD	DCMP deaminase	1.803	0.0206	212394	1635
34	ZNF100	Zinc finger protein 100	1.796	0.0418	1185658	163227
35	HTR2B	5-hydroxytryptamine (serotonin) receptor 2B	1.795	0.0383	272690	3357
36	HAL	Histidine ammonia-lyase	1.776	0.0134	345832	3034
37	SLC9A8	Solute carrier family 9 (sodium/hydrogen exchanger), member 8	1.772	0.0229	49515	23315
38	NOLC1	Nucleolar and coiled-body phosphoprotein 1	1.772	0.0436	843016	9221
39	IDE	Insulin-degrading enzyme	1.772	0.0139	48673	3416
40	RGS4	Regulator of G-protein signaling 4	1.755	0.0451	429349	5999
41	SLC4A1	Solute carrier family 4, anion exchanger, member 1	1.754	0.0225	295733	6521
42	LOC254559	Hypothetical protein LOC254559	1.741	0.0439	758200	254559
43	TAPBP	TAP binding protein (tapasin)	1.739	0.0336	242988	6892

44	CHD2	Chromodomain helicase DNA binding protein 2	1.738	0.0403	243580	1106
45	NIF3L1	NIF3 NGG1 interacting factor 3-like 1 (S. pombe)	1.734	0.0334	29648	60491
46	DOCK2	Dedicator of cytokinesis 2	1.713	0.0304	283444	1794
47	SMAD1	SMAD family member 1	1.707	0.0459	1927225	4086
48	MOBKL2B	MOB1, Mps One Binder kinase activator-like 2B	1.696	0.0404	48050	48050
49	CTNNA2	Catenin (cadherin-associated protein), alpha 2	1.695	0.0256	177772	1496
50	ESCO1	Establishment of cohesion 1 homolog 1 (S. cerevisiae)	1.694	0.0429	149744	114799
51	C8orf37	Chromosome 8 open reading frame 37	1.693	0.0390	726733	157657
52	CXCL1	Chemokine (C-X-C motif) ligand 1	1.692	0.0199	323238	2919
53	HSD17B3	Hydroxysteroid (17-beta) dehydrogenase 3	1.690	0.0011	758222	3293
54	KRTAP19-3	Keratin associated protein 19-3	1.675	0.0311	359635	337970
55	TMEM163	Transmembrane protein 163	1.675	0.0070	786580	81615
56	NUP43	Nucleoporin 43kDa	1.670	0.0150	774304	348995
57	OPA1	Optic atrophy 1 (autosomal dominant)	1.667	0.0178	209194	4976
58	UBE2I	Ubiquitin-conjugating enzyme E2I (UBC9 homolog, yeast)	1.666	0.0270	823883	7329
59	NCOR2	Nuclear receptor co-repressor 2	1.645	0.0092	150623	9612
60	TCP10L	T-complex 10 (mouse)-like	1.643	0.0284	230267	140290
61	C2ORF55	Chromosome 2 open reading frame 55	1.636	0.0232	714147	343990
62	C14ORF32	Chromosome 14 open reading frame 32	1.633	0.0107	40672	93487
63	RBM15	RNA binding motif protein 15	1.632	0.0253	280331	64783
64	NBS1	Nibrin	1.628	0.0377	153757	4683
65	CRHBP	Corticotropin releasing hormone binding protein	1.627	0.0377	701231	1393
66	ITIH4	Inter-alpha (globulin) inhibitor H4	1.622	0.0206	296123	3700
67	DICER1	Dicer1, Dcr-1 homolog (Drosophila)	1.618	0.0499	1388148	23405
68	GSTZ1	Glutathione transferase zeta 1	1.618	0.0412	769676	2954
69	FLJ20674	Hypothetical protein FLJ20674	1.616	0.0128	942520	54621
70	C7ORF10	Chromosome 7 open reading frame 10	1.613	0.0276	309499	79783
71	NUP43	Nucleoporin 43kDa	1.607	0.0192	146028	348995
72	FGF7	Fibroblast growth factor 7 (keratinocyte growth factor)	1.600	0.0137	365515	2252
73	PQLC1	PQ loop repeat containing 1	1.598	0.0284	741923	80148
74	JARID1A	Jumonji, AT rich interactive domain 1A	1.598	0.0446	1722714	5927
75	SEMA6A	Semaphorin 6A	1.596	0.0102	265522	57556
76	RFP	Tripartite motif-containing 27	1.589	0.0219	25366	5987
77	MYT1L	Myelin transcription factor 1-like	1.584	0.0348	41004	23040
78	TRIO	Triple functional domain (PTPRF interacting)	1.576	0.0082	2343489	7204
79	OXCT2	3-oxoacid CoA transferase 2	1.575	0.0448	1409412	64064
80	PLCB4	Phospholipase C, beta 4	1.567	0.0186	363352	5332
81	PRUNE2	Prune homolog 2 (Drosophila)	1.562	0.0303	230401	158471
82	ACTN1	Actinin, alpha 1	1.556	0.0176	122057	87
83	WDR41	WD repeat domain 41	1.556	0.0194	34585	55255
84	FKBP6	FK506 binding protein 6, 36kDa	1.550	0.0393	795736	8468
85	CYP19A1	Cytochrome P450, family 19, subfamily A, polypeptide 1	1.549	0.0480	2066371	1588
86	MTO1	Mitochondrial translation optimization 1 homolog	1.519	0.0261	266712	25821
87	SCC-112	PDS5, regulator of cohesion maintenance, homolog A	1.515	0.0325	503187	23244
88	CREBBP	CREB binding protein (Rubinstein-Taybi syndrome)	1.513	0.0118	417694	1387
89	LRFN3	Leucine rich repeat and fibronectin type III domain containing 3	1.505	0.0230	753968	79414
90	SYNJ1	Synaptojanin 1	1.501	0.0243	21984	8867
91	LAP3	Leucine aminopeptidase 3	1.497	0.0211	66476	51056
92	ZNF135	Zinc finger protein 135	1.486	0.0052	754529	7694
93	IFNAR2	Interferon (alpha, beta and omega) receptor 2	1.484	0.0282	359976	3455
94	ASB14	Ankyrin repeat and SOCS box-containing 14	1.483	0.0256	190215	142686

95	C20ORF19	Chromosome 20 open reading frame 19	1.482	0.0082	28086	55857
96	MRPL19	Mitochondrial ribosomal protein L19	1.481	0.0472	28989	9801
97	GABRB3	GABA A receptor, beta 3	1.481	0.0409	53022	2562
98	FLJ32784	Hypothetical protein FLJ32784	1.478	0.0368	2347699	127731
99	FLJ20619	Tetratricopeptide repeat domain 22	1.477	0.0488	235070	55001
100	CIDEB	Cell death-inducing DFFA-like effector b	1.468	0.0151	153653	27141
101	APXL	Shroom family member 2	1.467	0.0247	178818	357
102	KRT14	Keratin 14	1.462	0.0398	162681	3861
103	INHBC	Inhibin, beta C	1.455	0.0129	235069	3626
104	TMEM 63A	Transmembrane protein 63A	1.453	0.0378	1391653	9725
105	ME3	Malic enzyme 3, NADP(+)-dependent, mitochondrial	1.452	0.0273	35000	10873
106	IL6	Interleukin 6	1.451	0.0323	310406	3569
107	SMARCA3	Helicase-like transcription factor	1.451	0.0368	810974	6596
108	CIDEC	Cell death-inducing DFFA-like effector c	1.448	0.0220	155655	63924
109	RAPSN	Receptor-associated protein of the synapse	1.445	0.0253	759448	5913
110	SELP	Selectin P (CD62)	1.434	0.0343	108284	6403
111	RRM2B	Ribonucleotide reductase M2 B (TP53 inducible)	1.413	0.0294	172026	50484
112	ADMR	Adrenomedullin receptor	1.400	0.0335	1847208	11318
