# Supplementary material for: TEAD1 and c-Cbl are novel prostate basal cell markers that correlate with poor clinical outcome in prostate cancer
Source: Br J Cancer. 2008 Nov 11;99(11):1849–58. doi: 10.1038/sj.bjc.6604774 (PMC2600693; doi:10.1038/sj.bjc.6604774)
Supplement: Supplementary Table S2b [file 6604774x3.pdf]

Online only
Supplementary Table S2b: All genes up-regulated in luminal cells (ratio to basal cell expression)

Rank	Symbol	Name	Ratio	p-value	Clone ID	LLID
1	RPS11	Ribosomal protein S11	6.656	0.0057	172243	6205
2	VPS4B	Vacuolar protein sorting 4 homolog B	5.314	0.0186	150632	9525
3	VDAC3	Voltage-dependent anion channel 3	4.835	0.0360	796759	7419
4	SNAP25	Synaptosomal-associated protein, 25kDa	4.340	0.0050	969877	6616
5	PPP3CA	Protein phosphatase 3 catalytic subunit,alpha	4.260	0.0145	431296	5530
6	LRBA	LPS-responsive vesicle trafficking	4.254	0.0232	758404	987
7	ALDH9A1	Aldehyde dehydrogenase 9 family, A1	4.064	0.0159	36387	223
8	PDE6D	Phosphodiesterase 6D, cGMP-specific, rod, delta	3.944	0.0340	753199	5147
9	NBL1	Neuroblastoma, suppression of tumorigenicity 1	3.556	0.0162	365534	4681
10	FBP1	Fructose-1,6-bisphosphatase 1	3.486	0.0394	433253	2203
11	C7	Complement component 7	3.463	0.0067	757712	730
12	IPO11	Importin 11	3.440	0.0414	127826	51194
13	COL1A2	Collagen, type I, alpha 2	3.406	0.0221	271448	1278
14	NFASC	Neurofascin homolog (chicken)	3.350	0.0211	967492	23114
15	PFKM	Phosphofructokinase, muscle	3.291	0.0260	489626	5213
16	KBTBD11	Kelch repeat and BTB (POZ) domain cont 11	3.274	0.0164	384088	9920
17	PTHR1	Parathyroid hormone receptor 1	3.262	0.0334	1323328	5745
18	C1orf128	Chromosome 1 open reading frame 128	3.246	0.0408	175027	57095
19	ABLIM1	Actin binding LIM protein 1	3.238	0.0014	220294	3983
20	C1ORF14	Chromosome 1 open reading frame 14	3.212	0.0132	1409913	81626
21	SLC3A2	Solute carrier family 3, member 2	3.208	0.0292	150647	6520
22	COX6B1	Cytochrome c oxidase subunit Vib polypeptide 1	3.024	0.0018	172748	1340
23	NNMT	Nicotinamide N-methyltransferase	3.009	0.0467	85840	4837
24	GCH1	GTP cyclohydrolase 1 (dopa-responsive dystonia)	2.939	0.0088	783849	2643
25	HRASLS3	HRAS-like suppressor 3	2.935	0.0268	785293	11145
26	CAPZA1	Capping protein muscle Z-line, alpha 1	2.871	0.0364	785793	829
27	LRRC23	Leucine rich repeat containing 23	2.855	0.0066	302632	10233
28	TPI1	Triosephosphate isomerase 1	2.847	0.0311	855749	7167
29	MRCL3	Myosin regulatory light chain MRCL3	2.845	0.0093	296841	10627
30	C2ORF25	Chromosome 2 open reading frame 25	2.822	0.0085	207324	27249
31	CD47	CD47 molecule	2.781	0.0419	811819	961
32	GABBR1	Gamma-aminobutyric acid (GABA) B receptor, 1	2.756	0.0240	298231	2550
33	ENO1	Enolase 1, (alpha)	2.744	0.0227	723879	2023
34	GLG1	Golgi apparatus protein 1	2.738	0.0009	229579	2734
35	APOD	Apolipoprotein D	2.706	0.0265	159608	347
36	ENO2	Enolase 2 (gamma, neuronal)	2.682	0.0265	789147	2026
37	CDIPT	CDP-diacylglycerol-inositol 3-phosphatidyltransferase	2.680	0.0125	769901	10423
38	NDRG1	N-myc downstream regulated gene 1	2.649	0.0011	842863	10397
39	ALS2CR2	Amyotrophic lateral sclerosis 2chromosome region	2.645	0.0195	730342	55437
40	TCEAL6	Transcription elongation factor A (SII)-like 6	2.582	0.0146	2350376	158931
41	CAMKK2	Calcium/calmodulin-dep protein kinase kinase 2b	2.558	0.0357	209075	10645
42	FLJ23577	KPL2 protein	2.550	0.0027	1893348	79925
43	RPL24	Ribosomal protein L24	2.546	0.0267	795247	6152

44	SEC23IP	SEC23 interacting protein	2.544	0.0135	229464	11196
45	B2M	Beta-2-microglobulin	2.536	0.0368	141782	567
46	NKG7	Natural killer cell group 7 sequence	2.515	0.0098	71606	4818
47	MYH10	Myosin, heavy chain 10, non-muscle	2.494	0.0356	823886	4628
48	APLP2	Amyloid beta (A4) precursor-like protein 2	2.489	0.0408	366625	334
49	RRAGA	Ras-related GTP binding A	2.485	0.0040	34660	10670
50	ASTE1	Asteroid homolog 1 (Drosophila)	2.484	0.0452	768957	28990
51	CUL3	Cullin 3	2.483	0.0380	126815	8452
52	ARFGAP3	ADP-ribosylation factor GTPase activating protein 3	2.469	0.0191	259054	26286
53	SERPINE2	Serpin peptidase inhibitor, clade E member 2	2.449	0.0014	246722	5270
54	MGC99813	Similar to RIKEN cDNA A230078I05 gene	2.439	0.0140	124341	130612
55	NOL6	Nucleolar protein family 6 (RNA-associated)	2.439	0.0058	724138	65083
56	NYD-SP18	Family with sequence similarity 137, member A	2.408	0.0055	1638806	84691
57	ANAPC13	Anaphase promoting complex subunit 13	2.403	0.0132	502556	25847
58	ABLIM1	Actin binding LIM protein 1	2.386	0.0419	753692	3983
59	FLJ22318	Required for meiotic nuclear division 5 homolog B (S. cerevisiae)	2.344	0.0211	767827	64777
60	CKLFSF4	CKLF-like MARVEL transmembrane domain containing 4	2.335	0.0017	279411	146223
61	BAZ1B	Bromodomain adjacent to zinc finger domain, 1B	2.331	0.0232	366041	9031
62	KLHL5	Kelch-like 5 (Drosophila)	2.328	0.0123	704793	51088
63	DSCR2	Down syndrome critical region gene 2	2.323	0.0090	377539	8624
64	MYLK	Myosin, light chain kinase	2.320	0.0428	841308	4638
66	SLC24A1	Solute carrier family 24 (sodium/potassium/calcium exchanger)	2.308	0.0101	360741	9187
67	VPS35	Vacuolar protein sorting 35 homolog (S. cerevisiae)	2.303	0.0353	281010	55737
68	SPTBN1	Spectrin, beta, non-erythrocytic 1	2.303	0.0369	276621	6711
69	AMFR	Autocrine motility factor receptor	2.301	0.0039	753897	267
70	H2AFV	H2A histone family, member V	2.300	0.0204	121452	94239
71	TLE2	Transducin-like enhancer of split 2 (E(sp1) homolog, Drosophila)	2.293	0.0147	1473131	7089
72	MAP3K7IP2	Mitogen-activated protein kinase kinase kinase 7 int prot 2	2.281	0.0249	1568205	23118
73	GA17	Eukaryotic translation initiation factor 3, subunit M	2.280	0.0204	124733	10480
74	UGP2	UDP-glucose pyrophosphorylase 2	2.274	0.0241	486436	7360
75	FGFR2	Fibroblast growth factor receptor 2	2.273	0.0097	26871	2263
76	CDKN1B	Cyclin-dependent kinase inhibitor 1B (p27, Kip1)	2.266	0.0236	854668	1027
77	SDHC	Succinate dehydrogenase complex, subunit C,	2.262	0.0026	366132	6391
78	SELT	Selenoprotein T	2.258	0.0022	728216	51714
79	IL17RC	Interleukin 17 receptor C	2.256	0.0494	755424	84818
80	DECR1	2,4-dienoyl CoA reductase 1, mitochondrial	2.254	0.0464	320618	1666
81	TUBE1	Tubulin, epsilon 1	2.253	0.0220	489765	51175
82	COPS6	COP9 constitutive photomorphogenic homolog subunit 6	2.242	0.0078	208431	10980
83	KIAA0376	SPECC1-like	2.237	0.0269	32472	23384
84	WDR26	WD repeat domain 26	2.234	0.0025	268250	80232
85	EIF4G1	Eukaryotic translation initiation factor 4 gamma, 1	2.209	0.0213	25988	1981
86	COQ6	Coenzyme Q6 homolog, monooxygenase (S. cerevisiae)	2.198	0.0229	84880	51004
87	C4A	Complement component 4B (Childo blood group)	2.192	0.0078	26063	721
88	RANBP6	RAN binding protein 6	2.192	0.0123	43819	26953
89	OSBPL10	Oxysterol binding protein-like 10	2.185	0.0084	151597	114884
90	CIB1	Calcium and integrin binding 1 (calmyrin)	2.175	0.0165	841679	10519
91	SGTA	Small glutamine-rich tetratricopeptide repeat-containing, alpha	2.165	0.0374	174620	6449
92	SVIP	Small VCP/p97-interacting protein	2.157	0.0403	768573	258010
93	SIAT7A	ST6 -N-acetylgalactosaminide alpha-2,6-sialyltransferase 1	2.148	0.0258	1391774	55808
94	MGC10854	Trichoplein, keratin filament binding	2.144	0.0056	1534055	84260
95	PHB	Prohibitin	2.143	0.0270	357448	5245

96	LTA4H	Leukotriene A4 hydrolase	2.140	0.0222	814095	4048
97	ATP5I	ATP synthase, mitochondrial F0 complex, subunit E	2.139	0.0467	782439	521
98	TPPP	Tubulin polymerization promoting protein	2.136	0.0452	172895	11076
99	DCN	Decorin	2.133	0.0254	1643268	1634
100	BLP1	TM2 domain containing 2	2.132	0.0394	42822	83877
101	COX11P	COX11 homolog, cytochrome c oxidase assembly protein (yeast)	2.121	0.0394	28259	1353
102	MAL	Mal, T-cell differentiation protein	2.118	0.0287	31154	4118
103	POLR2G	Polymerase (RNA) II (DNA directed) polypeptide G	2.109	0.0199	219635	5436
104	SND1	Staphylococcal nuclease and tudor domain containing 1	2.109	0.0429	361048	27044
105	PTP4A1	Protein tyrosine phosphatase type IVA, member 1	2.108	0.0135	26097	7803
106	MRLC2	Myosin regulatory light chain MRLC2	2.102	0.0013	841498	103910
107	CAPNS1	Calpain, small subunit 1	2.091	0.0409	174796	826
108	SERP1	Stress-associated endoplasmic reticulum protein 1	2.082	0.0328	302955	27230
109	SHFM1	Split hand/foot malformation (ectrodactyly) type 1	2.075	0.0156	346613	7979
110	LOC653354	Similar to amrmadillo repeat containing, X-linked 6 -like	2.069	0.0359	811064	653354
111	DDX3Y	DEAD (Asp-Glu-Ala-Asp) box polypeptide 3, Y-linked	2.068	0.0125	782679	8653
112	SCNN1A	Sodium channel, nonvoltage-gated 1 alpha	2.053	0.0058	810873	6337
113	CDKN1A	Cyclin-dependent kinase inhibitor 1A (p21, Cip1)	2.048	0.0207	166273	1026
114	C2ORF29	Chromosome 2 open reading frame 29	2.046	0.0486	146133	55571
115	FRG1	FSHD region gene 1	2.034	0.0353	795919	2483
116	SPRYD4	SPRY domain containing 4	2.022	0.0442	120750	283377
117	PRKCI	Protein kinase C, iota	2.020	0.0252	1882357	5584
118	NUMA1	Nuclear mitotic apparatus protein 1	2.018	0.0421	154568	4926
119	ECHDC2	Enoyl Coenzyme A hydratase domain containing 2	2.007	0.0010	198208	55268
120	SPRY1	Sprouty homolog 1, antagonist of FGF signaling	2.003	0.0150	377468	10252
121	KLF11	Kruppel-like factor 11	1.982	0.0096	110959	8462
122	KIAA0152	KIAA0152	1.980	0.0134	111653	9761
123	EIF3S9	Eukaryotic translation initiation factor 3, subunit B	1.972	0.0030	768052	8662
124	HSPA1L	Heat shock 70kDa protein 1-like	1.961	0.0455	35091	3305
125	PELI1	Pellino homolog 1 (Drosophila)	1.958	0.0259	344442	57162
126	RAB6IP1	RAB6 interacting protein 1	1.957	0.0360	364434	23258
127	MSN	Moesin	1.950	0.0004	131362	4478
128	CRSP7	Mediator complex subunit 26	1.946	0.0132	276714	9441
129	ATRN	Attractin	1.941	0.0016	40539	8455
131	SATB1	SATB homeobox 1	1.939	0.0236	1556983	6304
132	PTPRU	Protein tyrosine phosphatase, receptor type, U	1.915	0.0244	744800	10076
133	BNC2	Basonuclin 2	1.913	0.0256	269733	54796
134	IQGAP2	IQ motif containing GTPase activating protein 2	1.903	0.0356	321386	10788
135	MICAL3	Microtubule associated monoxygenase	1.902	0.0318	201056	57553
136	NRN1	Neuritin 1	1.902	0.0257	38531	51299
137	SNRK	SNF related kinase	1.902	0.0287	342720	54861
138	KPNA6	Karyopherin alpha 6 (importin alpha 7)	1.898	0.0068	151012	23633
139	ARHN	Rho family GTPase 2	1.890	0.0285	757139	8153
140	BBOX1	Butyrobetaine (gamma), 2-oxoglutarate dioxygenase 1	1.889	0.0211	34218	8424
141	DLEU2	Deleted in lymphocytic leukemia, 2	1.882	0.0290	296248	8847
142	GNPAT	Glyceronephosphate O-acyltransferase	1.882	0.0319	841287	8443
143	VIPR1	Vasoactive intestinal peptide receptor 1	1.880	0.0109	110090	7433
144	DSG2	Desmoglein 2	1.879	0.0323	842971	1829
145	SYK	Spleen tyrosine kinase	1.876	0.0241	897822	6850
146	TRIP4	Thyroid hormone receptor interactor 4	1.871	0.0290	785334	9325
147	ZNFN1A5	IKAROS family zinc finger 5 (Pegasus)	1.870	0.0081	128274	64376

148	ARL10C	ADP-ribosylation factor-like 8B	1.869	0.0286	39677	55207
149	ANXA5	Annexin A5	1.862	0.0236	786680	308
150	AVEN	Apoptosis, caspase activation inhibitor	1.856	0.0048	377368	57099
151	THRAP1	Mediator complex subunit 13	1.854	0.0296	136451	9969
152	TDG	Thymine-DNA glycosylase	1.843	0.0223	823614	6996
153	PDLIM1	PDZ and LIM domain 1 (elfin)	1.827	0.0277	298984	9124
154	HNRPU	Heterogeneous nuclear ribonucleoprotein U	1.819	0.0128	22694	3192
155	SHMT1	Serine hydroxymethyltransferase 1 (soluble)	1.811	0.0302	39798	6470
156	POLDIP3	Polymerase (DNA-directed), delta interacting protein 3	1.810	0.0307	35383	84271
157	MGC15882	Chromosome 1 open reading frame 94	1.805	0.0260	1525744	84970
158	MGC33510	Alcohol dehydrogenase, iron containing, 1	1.802	0.0215	41398	137872
159	FHL2	Four and a half LIM domains 2	1.796	0.0421	324636	2274
160	ZCCHC14	Zinc finger, CCHC domain containing 14	1.794	0.0436	310703	23174
161	CADPS	Ca2+-dependent secretion activator	1.794	0.0066	39794	8618
162	TXNL4A	Thioredoxin-like 4A	1.793	0.0373	323317	10907
163	KCNH2	Potassium voltage-gated channel, subfamily H (eag-related)	1.790	0.0156	125570	3757
164	PLA2R1	Phospholipase A2 receptor 1, 180kDa	1.788	0.0129	114764	22925
165	SIAT4A	Hypothetical protein LOC286167	1.786	0.0334	293391	286167
166	N-PAC	Cytokine-like nuclear factor n-pac	1.785	0.0158	343629	84656
167	SMARCA4	SWI/SNF related, actin dependent regulator of chromatin,	1.783	0.0292	173169	6597
168	WAC	WW domain containing adaptor with coiled-coil	1.781	0.0237	21790	51322
169	OSTM1	Osteopetrosis associated transmembrane protein 1	1.772	0.0348	30038	28962
170	LOC728974	Similar to peptidylprolyl isomerase A isoform 1	1.768	0.0043	241900	728974
171	XTP2	BAT2 domain containing 1	1.760	0.0222	123408	23215
172	PTGES	Prostaglandin E synthase	1.749	0.0126	754378	9536
173	KIAA0232	KIAA0232	1.746	0.0451	278663	9778
174	C3orf59	Chromosome 3 open reading frame 59	1.738	0.0045	33196	151963
175	THSD1P	Thrombospondin, type I, domain containing 1 pseudogene	1.735	0.0056	108374	374500
176	EIF4ENIF1	Eukaryotic translation initiation factor 4E nuclear import factor 1	1.735	0.0425	1393138	56478
177	PSME1	Proteasome (prosome, macropain) activator subunit 1	1.733	0.0054	25849	5720
178	MYO9A	Myosin IXA	1.733	0.0164	727977	4649
179	PPP1R12B	Protein phosphatase 1, regulatory (inhibitor) subunit 12B	1.724	0.0426	307174	4660
180	A2M	Hypothetical protein LOC144571	1.724	0.0137	25715	144571
181	ARL2BP	ADP-ribosylation factor-like 2 binding protein	1.720	0.0335	37264	23568
182	PPP1R9A	Protein phosphatase 1, regulatory (inhibitor) subunit 9A	1.711	0.0343	942306	55607
183	P29	SYF2 homolog, RNA splicing factor (S. cerevisiae)	1.709	0.0417	49546	25949
184	SURF4	Surfeit 4	1.708	0.0290	109423	6836
185	PRRX1	Paired related homeobox 1	1.708	0.0070	364554	5396
186	PPP1R15B	Protein phosphatase 1, regulatory (inhibitor) subunit 15B	1.705	0.0309	814834	84919
187	API5	Apoptosis inhibitor 5	1.699	0.0315	471597	8539
188	HIST1H4C	Histone cluster 1, H4c	1.699	0.0160	1461138	8364
189	PSG7	Pregnancy specific beta-1-glycoprotein 7	1.697	0.0299	292935	5676
190	SCAP	SREBF chaperone	1.696	0.0396	22109	22937
191	HNRPUL1	Heterogeneous nuclear ribonucleoprotein U-like 1	1.693	0.0486	810395	11100
192	UBE2B	Ubiquitin-conjugating enzyme E2B (RAD6 homolog)	1.687	0.0189	898138	7320
193	MTRF1	Mitochondrial translational release factor 1	1.686	0.0341	823638	9617
194	RNF167	Ring finger protein 167	1.679	0.0355	307964	26001
195	RNF144	Ring finger protein 144A	1.677	0.0054	33573	9781
196	MGAM	Maltase-glucoamylase (alpha-glucosidase)	1.676	0.0388	1502206	8972
197	ATP6V1F	ATPase, H+ transporting, lysosomal 14kDa, V1 subunit F	1.664	0.0462	126278	9296
198	PLCB1	Phospholipase C, beta 1 (phosphoinositide-specific)	1.664	0.0075	360865	23236

199	PRM2	Protamine 2	1.663	0.0051	1600243	5620
200	ABAT	4-aminobutyrate aminotransferase	1.660	0.0139	171774	18
201	SCAMP2	Secretory carrier membrane protein 2	1.655	0.0181	769846	10066
202	ASAHL	N-acylsphingosine amidohydrolase (acid ceramidase)-like	1.648	0.0085	1682030	27163
203	NCDN	Neurochondrin	1.643	0.0153	27521	23154
204	MTHFR	Chromosome 1 open reading frame 167	1.640	0.0385	1894346	284498
205	SOX6	Chromosome 11 open reading frame 58	1.633	0.0041	205464	10944
206	KIAA0415	Forkhead box K1	1.630	0.0210	756419	221937
207	RHOG	Ras homolog gene family, member G (rho G)	1.626	0.0385	39541	391
208	ATP11B	ATPase, Class VI, type 11B	1.625	0.0281	199286	23200
210	RDBP	RD RNA binding protein	1.623	0.0016	509484	7936
211	SEC23B	Sec23 homolog B (S. cerevisiae)	1.618	0.0190	743804	10483
212	MARCH11	Membrane-associated ring finger (C3HC4) 11	1.615	0.0466	1883888	441061
213	MGC14327	Hypothetical protein MGC14327	1.613	0.0372	277570	94107
214	DC12	Chromosome 3 open reading frame 37	1.613	0.0333	40233	56941
215	ZC3HAV1	Zinc finger CCCH-type, antiviral 1	1.612	0.0398	200187	56829
216	PSD2	Pleckstrin and Sec7 domain containing 2	1.610	0.0079	38794	84249
217	OXCT1	3-oxoacid CoA transferase 1	1.605	0.0170	28469	5019
218	TCERG1	Transcribed locus	1.602	0.0472	1251292	125129<br>2
219	TMEM31	Transmembrane protein 31	1.597	0.0035	742547	203562
220	CESK1	Chaperonin containing TCP1, subunit 8 (theta)-like 2	1.597	0.0283	1586695	150160
221	PPM2C	Protein phosphatase 2C, magnesium-dependent, catalytic subunit	1.591	0.0194	174304	54704
222	TSPAN2	Tetraspanin 2	1.590	0.0239	486605	10100
223	HIVEP1	Human immunodeficiency virus type I enhancer binding protein 1	1.589	0.0178	267094	3096
224	PPP1R11	Protein phosphatase 1, regulatory (inhibitor) subunit 11	1.588	0.0455	810986	6992
225	SNRPC	Small nuclear ribonucleoprotein polypeptide C	1.583	0.0491	724387	6631
226	CCNK	Cyclin K	1.581	0.0173	1686512	8812
227	BRD8	Bromodomain containing 8	1.574	0.0284	815287	10902
228	CDC14A	CDC14 cell division cycle 14 homolog A (S. cerevisiae)	1.568	0.0128	293403	8556
229	EML1	Echinoderm microtubule associated protein like 1	1.557	0.0137	25163	2009
230	HT007	Transmembrane protein 126B	1.555	0.0257	269998	55863
231	LOC723809	LOC723809	1.555	0.0294	2383496	723809
232	KIAA0310	SEC16 homolog A (S. cerevisiae)	1.553	0.0180	809944	9919
233	C13orf15	Chromosome 13 open reading frame 15	1.551	0.0056	1520384	28984
234	AKT3	V-akt murine thymoma viral oncogene homolog 3	1.550	0.0196	34539	10000
235	ITGB3BP	Integrin beta 3 binding protein (beta3-endonexin)	1.549	0.0029	298020	23421
236	EWSR1	Ewing sarcoma breakpoint region 1	1.543	0.0473	108304	2130
237	FLJ32942	Family with sequence similarity 112, member B	1.542	0.0408	109045	121355
238	TPX2	TPX2, microtubule-associated, homolog (Xenopus laevis)	1.534	0.0272	232837	22974
239	KIAA0056	Non-SMC condensin II complex, subunit D3	1.533	0.0312	770066	23310
240	PCGF5	Polycomb group ring finger 5	1.533	0.0474	2009704	84333
241	RAB9P40	Rab9 effector protein with kelch motifs	1.527	0.0083	173344	10244
242	HDHD2	Haloacid dehalogenase-like hydrolase domain containing 2	1.527	0.0251	28643	84064
243	PDK1	Pyruvate dehydrogenase kinase, isozyme 1	1.522	0.0485	1645668	5163
244	NPAS2	Transcribed locus	1.522	0.0364	147776	147776
245	ADAMTSL1	ADAMTS-like 1	1.520	0.0263	752797	92949
246	ZNF228	Zinc finger protein 228	1.516	0.0419	322683	7771
247	ADAMTS17	ADAM metallopeptidase with thrombospondin type 1 motif, 17	1.513	0.0179	364454	170691
248	IVL	Involucrin	1.509	0.0194	2449565	3713
249	NT5DC3	5'-nucleotidase domain containing 3	1.508	0.0262	41892	51559

250	SLC6A1	Solute carrier family 6 (neurotransmitter transporter, GABA),1	1.503	0.0270	172250	6529
251	PLA2G12B	Phospholipase A2, group XIIB	1.502	0.0270	297107	84647
252	RNASEL	Ribonuclease L (2',5'-oligoisoadenylate synthetase-dependent)	1.500	0.0216	321167	6041
253	RTN4	Reticulon 4	1.497	0.0287	38934	57142
254	RANBP3	RAN binding protein 3	1.496	0.0466	490045	8498
255	XAB2	XPA binding protein 2	1.494	0.0478	28448	56949
256	TUBA1	Tubulin, alpha 4a	1.492	0.0363	34579	7277
257	TOLLIP	Toll interacting protein	1.489	0.0449	768500	54472
258	GPR19	G protein-coupled receptor 19	1.488	0.0388	45231	2842
259	SEC3L1	Exocyst complex component 1	1.486	0.0408	1251877	55763
260	FHL5	Four and a half LIM domains 5	1.482	0.0304	1409893	9457
261	SFRS11	Splicing factor, arginine/serine-rich 11	1.478	0.0171	1390404	9295
262	PABPC5	Poly(A) binding protein, cytoplasmic 5	1.470	0.0407	878759	140886
263	PLN	Phospholamban	1.468	0.0226	773771	5350
264	UREB1	HECT, UBA and WWE domain containing 1	1.460	0.0437	297011	10075
265	ATAD1	ATPase family, AAA domain containing 1	1.459	0.0151	26005	84896
266	CSPG3	Neurocan	1.427	0.0413	41261	1463
267	LOC644339	Similar to ankyrin repeat domain 20A	1.423	0.0391	134742	644339
