# Supplementary material for: TEAD1 and c-Cbl are novel prostate basal cell markers that correlate with poor clinical outcome in prostate cancer
Source: Br J Cancer. 2008 Nov 11;99(11):1849–58. doi: 10.1038/sj.bjc.6604774 (PMC2600693; doi:10.1038/sj.bjc.6604774)
Supplement: Supplementary Table S3 [file 6604774x4.pdf]

Online Only

Supplementary Table S3: Functional categories for basal and luminal expressed genes

Functional Categories	P-Value	Example genes
**BASAL LAYER**		
Positive regulation of physiological process	0.0381	*IL6, VEGFC, CBL, JARID1A, CIDEB, CIDEC,*
Receptor binding	0.0479	*TAPBP, IL6, VEGFC, DOCK2, CXCL1, INHBC, NCOR2,*
Positive regulation of biological process	0.0480	*IL6, VEGFC, CBL, JARID1A, CIDEB, CIDEC, HTR2B,*
Protein binding	0.0008	*DOCK2, ITGAV, CRHBP, DICER1, CXCL1, TEAD1, IL6, CBL, RFP*
RNA processing	0.0127	*MTO1, C1ORF19, DICER1, SNRPB2, NOLC1, TXNL4A, SFRS1,*
Enzyme regulator activity	0.0456	*TRIO, ITIH4, DOCK2, ARL1, A2M, CXCL1, RGS4, RGS11,*
RNA metabolism	0.0343	*MTO1, C1ORF19, DICER1, SNRPB2, NOLC1, TXNL4A, SFRS1,*
Protein complex assembly	0.0170	*TAPBP, A2M, CREBBP, TXNL4A, SFRS1, KIFAP3,*
Positive regulation of cellular process	0.0231	*IL6, VEGFC, CBL, JARID1A, CIDEB, CIDEC, HTR2B,*
Positive regulation of cellular physiological process	0.0322	*IL6, VEGFC, CBL, JARID1A, CIDEB, CIDEC,*
Helicase activity	0.0388	*C9ORF102, DICER1, SMARCA3, CHD2,*
Growth factor activity	0.0131	*IL6, VEGFC, CXCL1, INHBC,*
Ligase activity, forming carbon-nitrogen bonds	0.0367	*RAPSN, UBE2I, CBL, RFP, SMARCA3, HAL,*
Cellular process	0.0377	*GSTZ1, ITGAV, CXCL1, TEAD1, NOLC1, IFNAR2, IL6, C1ORF19, FKBP6*
**LUMINAL LAYER**		
Calmodulin binding	0.0030	*EWSR1, SPTBN1, CAMKK2, MYH10, IQGAP2, PPP3CA, MYLK,*
Nucleotide binding	0.0346	*SYK, PDK1, HNRPU, RDBP, SNRK, PABPC5, ATAD1, PRKCI, FGFR2, SFRS11*
Regulation of biological process	0.0401	*CDKN1B, NBL1, CDKN1A, SNAP25, SPRY1 ITGB3BP ENO1, CUL3, SOX6, PHB*
Protein transporter activity	0.0162	*ARFGAP3, A2M, KPNA6, EIF4ENIF1, IPO11,*
Hydrolase activity	0.0230	*PLA2G12B, ATAD1, SHFM1, ASAHL, SND1, HDHD2, CDC14A, ASTE1 ENO1*
Protein transport	0.0488	*A2M, ARFGAP3, PRKCI, VPS35, SEC23B, CADPS, SCAMP2, KPNA6, RANBP6, RANBP3*
Ion binding	0.0496	*PLA2G12B, CDKN1A, RNF144, BNC2, AMFR, ZNF228, RNF167, ENO1, ENO2*
Cell cycle	0.0348	*CCNK, CDKN1B, CUL3, CDC14A, NBL1, PLCB1, PRM2, HRASLS3, CDKN1A, PHB*
Cellular physiological process	0.0015	*NBL1, CDKN1A, ITGB3BP, SYK, CCNK, CUL3, FGFR2, SOX6, SNAP25, ENO1, PHB*
Alcohol metabolism	0.0110	*PDK1, TPI1, UGP2, N-PAC, ALDH9A1, PFKM, ENO1, FBP1, ENO2, SCAP,*
Cellular localization	0.0463	*TUBA1, VPS4B, A2M, ARFGAP3, PRKCI, MAL, VPS35, SEC23B, TUBE1, SCAMP2*
Protein localization	0.0368	*ARFGAP3, PRKCI, MAL, VPS35, SEC23B, CADPS, SCAMP2, KPNA6, RANBP6, RANBP3*
Enzyme regulator activity	0.0096	*CDKN1B, ARL2BP, SERPINE2, PLN, ARFGAP3, IQGAP2, PPP1R12B*
Protein binding	0.0000	*CDKN1B, PLN, NBL1, CDKN1A, SNAP25, ITGB3BP, RPL24, SYK, CCNK, IQGAP2*
Transcriptional activator activity	0.0046	*PHB, SMARCA4, TRIP4, THRAP1, BRD8, FHL2, TCERG1, CRSP7, PRRX1,*
Carbohydrate metabolism	0.0317	*PDK1, UGP2, TPI1, N-PAC, PFKM, SDHC, OXCT1, TDG, SLC3A2, MGAM*
Transcription factor binding	0.0242	*SMARCA4, TRIP4, THRAP1, BRD8, FHL2, TCERG1, CRSP7, SND1, PRRX1,*
Actin binding	0.0231	*SPTBN1, MYH10, ABLIM1, IQGAP2, CAPZA1, MSN, KLHL5, PPP1R9A,*
Cell proliferation	0.0384	*C2ORF29, SYK, CDKN1B, CAPNS1, CUL3, CDC14A, CDKN1A, PHB, KLF11, RHOG*
Muscle contraction	0.0377	*KCNH2, PLN, VIPR1, PPP1R12B, MRCL3, MRLC2, C4A,*
Cell organization and biogenesis	0.0024	*A2M, H2AFV, PRKCI, FGFR2, SOX6, PHB, SPTBN1, BRD8, ABLIM1, SATB1*
Cell motility	0.0101	*SYK, CSPG3, AMFR, SERPINE2, CAPZA1, RTN4, MSN, PSG7, TSPAN2,*
Organic acid metabolism	0.0488	*ABAT, BBOX1, TPI1, LTA4H, GCH1, PTGES, SHMT1, ENO1, GNPAT, MTHFR*

In order to identify key biological processes, basal and luminal gene lists were individually entered into the web based ontology program DAVID (http://david.abcc.ncifcrf.gov/). This program categorises genes based on their biological or molecular function and assigns a p-value to each category to indicate its overall significance. Filtering on a p-value less than or equal to 0.05 returned 14 basal cell categories and 23 luminal categories. Table S3 shows the final list of categories for both populations, including up to 10 example genes from each category and the category p-value.
