# Supplementary material for: TEAD1 and c-Cbl are novel prostate basal cell markers that correlate with poor clinical outcome in prostate cancer
Source: Br J Cancer. 2008 Nov 11;99(11):1849–58. doi: 10.1038/sj.bjc.6604774 (PMC2600693; doi:10.1038/sj.bjc.6604774)
Supplement: Supplementary Table S4 [file 6604774x5.pdf]

Online Only

Supplementary Table 4

Primer Sequences

Product name	Forward primer	Reverse primer	Product Size (bp)
ITGAV	AGGCAGATGGCAAAGGAGTA	TGCTCCCTTTTGCTTGAGTT	83
CBL	GGGGGTCACTGTTCATCACT	TGTTTGGGACCATAAACACA	75
TEAD1	TTCCACCAAAGTTTGCTCCT	GCCATTCTCAAACCTTGCAT	76
SNAP25	CTGCTCGTGTAGTGGACGAA	ATTTTCTCGGGCATCATTTG	83
GAPDH	GGGAAGCTTGTCATCAATGG	TTGATTTTGGAGGGATCTCG	65
