# Supplementary material for: TEAD1 and c-Cbl are novel prostate basal cell markers that correlate with poor clinical outcome in prostate cancer
Source: Br J Cancer. 2008 Nov 11;99(11):1849–58. doi: 10.1038/sj.bjc.6604774 (PMC2600693; doi:10.1038/sj.bjc.6604774)
Supplement: Supplementary materials and methods [file 6604774x6.doc]

# Online only

# Supplementary materials and methods

**Gene expression profiling and analysis**

cDNA microarray analysis using the Cancer Research UK Human Whole Genome-wide Array v1.0.0 (32K) ([http://www.crukdmf.icr.ac.uk](http://www.crukdmf.icr.ac.uk/)) was used to examine gene expression differences.

Analysis was performed using GeneSpring version 7.2 (Agilent Technologies, Waldbronn, Germany). Array data was normalised to LOWESS (locally weighted least squares) and to ‘per gene to median normalisation’. A Welch t-test was performed on normalised data between the 5 basal and 5 luminal cell samples. The data was filtered using a *p*-value of 0.05 or less and a Benjamin and Hochberg multiple testing correction (a false discovery rate) of 5% applied. Data fulfilling these criteria were entered into hierarchical cluster analysis to display basal and luminal differences. These data were further filtered according to expression level, using a cut-off of 1.4 fold (up or down) in at least 3 out of the 5 samples. All raw microarray data was formatted for ‘Gene Expression Omnibus’ (GEO) (<http://www.ncbi.mln.nih.gov/geo>) to comply with Minimum Information About a Microarray Experiment (MIAME) and Microarray Gene Expression Database (MGED) group standards.

**Database for Annotation Visualization and Integrated Discovery (DAVID) Interrogation**

Basal and luminal genes that passed all of the described filtering criteria (including the fold change cut off) were entered into the Gene Functional Annotation Tool available at the DAVID website (<http://david.abcc.ncifcrf.gov/>) using their official gene symbols. Gene ontology options GOTERM_BP_ALL and GOTERM_MF_ALL were selected and a functional annotation chart generated. A maximum *p*-value of 0.05 was chosen to select only significant categories.

**Primer design**

Primers were designed using Primer3 software and were purchased from Invitrogen, sequences can be found in supplementary data. All primers spanned an intron/exon boundary, annealed away from the extreme ends of transcripts and generated short amplicons (50-80bp). Two rounds of PCR were required to detect transcripts. Primer details are given in Supplementary Table 4. PCR products were resolved on 3% agarose gels.

The following cycling conditions were used:

**First round 20-50ng cDNA input:**

(94^o^C 1 min) x1;

(94 ^o^C 30s, 60 ^o^C 30s*, 72 ^o^C 30s) x10 *decrementing 1^o^C per cycle.;

(94 ^o^C 30s, 52 ^o^C 30s, 72 ^o^C 30s) x10.

**Second round using 10% of first round PCR product:**

(94 ^o^C 1 min) x1;

(94 ^o^C 30s, 60 ^o^C 30s*, 72 ^o^C 30s) x10 *decrementing 1^o^C per cycle;

(94 ^o^C 30s, 52 ^o^C 30s, 72 ^o^C 30s) x30;

(72 ^o^C 10 min) x1
